# Identification and Target-Modification of SL-BBI: A Novel Bowman–Birk Type Trypsin Inhibitor from *Sylvirana latouchii*

**DOI:** 10.3390/biom10091254

**Published:** 2020-08-28

**Authors:** Xi Chen, Dong Chen, Linyuan Huang, Xiaoling Chen, Mei Zhou, Xinping Xi, Chengbang Ma, Tianbao Chen, Lei Wang

**Affiliations:** 1 Natural Drug Discovery Group, School of Pharmacy, Queen’s University Belfast, Belfast BT9 7BL, UK; xchen27@qub.ac.uk (X.C.); dchen03@qub.ac.uk (D.C.); x.chen@qub.ac.uk (X.C.); m.zhou@qub.ac.uk (M.Z.); x.xi@qub.ac.uk (X.X.); t.chen@qub.ac.uk (T.C.); l.wang@qub.ac.uk (L.W.); 2 School of Life Sciences and Technology, China Pharmaceutical University, Nanjing 211198, China; huangly@cpu.edu.cn

**Keywords:** natural molecules, Bowman–Birk type inhibitor, ranacyclin, antimicrobial, anticancer

## Abstract

The peptides from the ranacyclin family share similar active disulphide loop with plant-derived Bowman–Birk type inhibitors, some of which have the dual activities of trypsin inhibition and antimicrobial. Herein, a novel Bowman–Birk type trypsin inhibitor of the ranacyclin family was identified from the skin secretion of broad-folded frog (*Sylvirana latouchii*) by molecular cloning method and named as SL-BBI. After chemical synthesis, it was proved to be a potent inhibitor of trypsin with a Ki value of 230.5 nM and showed weak antimicrobial activity against tested microorganisms. Modified analogue K-SL maintains the original inhibitory activity with a Ki value of 77.27 nM while enhancing the antimicrobial activity. After the substitution of active P1 site to phenylalanine and P2′ site to isoleucine, F-SL regenerated its inhibitory activity on chymotrypsin with a Ki value of 309.3 nM and exhibited antiproliferative effects on PC-3, MCF-7 and a series of non-small cell lung cancer cell lines without cell membrane damage. The affinity of F-SL for the β subunits in the yeast 20S proteasome showed by molecular docking simulations enriched the understanding of the possible action mode of Bowman–Birk type inhibitors. Further mechanistic studies have shown that F-SL can activate caspase 3/7 in H157 cells and induce apoptosis, which means it has the potential to become an anticancer agent.

## 1. Introduction

Serine protease inhibitors can regulate a variety of physiological reactions in the body by preventing unnecessary proteolytic functions. More than 1500 natural serine protease inhibitors from animal tissues and bodily fluids, plants, bacteria, fungi and viruses have been found at present. Among them, Bowman–Birk inhibitor (BBI) is a peptide family rich in cysteine and supported by seven intramolecular disulphide bonds, with dual inhibitory activity of trypsin and chymotrypsin due to its two independent reaction ring exposure at the molecular terminal [1]. The more concise BBI sequences are usually within 20 amino acids and contain an 11-residue active reaction loop, almost all of which are present in amphibian skin secretions and were generalised as the ranacyclin family [2].

Substantial evidence suggests that trypsin inhibitor peptides own prominent activity in protease inhibitory [3], antimicrobial [4] and anticarcinogenic [5,6,7,8,9], few of which are even considered as potent immunomodulators with hemopoietic actions [10]. Ranacyclin is the unique family of serine protease inhibitors known from amphibian skin secretions and usually has the dual activities of trypsin inhibition and antibacterial [2,11] to combat the invasion of foreign invasive pathogens. Additionally, the proliferation, migration, spread and invasion of cancer cells are complex pathological processes involving multiple proteolytic enzymes [12]. Although the specific mechanism of BBI as an anticancer factor is unclear, protease inhibition has been shown to reverse the development in the early stages of tumorigenesis. The broad-spectrum anti-proliferation effects by BBIs act on cancer types such as lung [5,13], prostate [5,8], colon [6], breast [9,14,15], and oral [16]. It is particularly worth mentioning that the few side effects [5,13,15,17] and radioprotection [18,19] to normal mammalian cells establish the cancer chemoprevention properties of such peptides. Many types of researched protease inhibitors also have gained huge significance as potential pharmacological tools in cancer treatment [15,20,21,22] may be through inhibiting the activity of proteasome 20S and promotes apoptosis through ROS-induced mitochondrial damage following proteasome inhibition [14]. Furthermore, BBI can also affect the expression of certain oncogenes, the activity of certain proteolytic reactions (increased activity of this response in tissues exposed to the cancer-induced factor) and reverse the initial phase of malignant transformation induced by radiation and chemical carcinogen [23]. Inhibitors of BBI families have been confirmed to bind to classical bradykinin and its analogues as a protective pathway to against the cleavage of plasmatic serine proteases, leading to an increase in the half-life of bradykinin [24].

In this research, a novel Bowman–Birk type protease inhibitor named SL-BBI was purified and characterised from the skin secretion of *Sylvirana latouchii*. While the biofunction of SL-BBI was estimated, we carried out targeted modifications to promote the antimicrobial efficacy based on maintaining trypsin inhibition and increase anticancer activity, respectively. Both analogues, K-SL and F-SL, produced the expected promotion after functional assessment.

## 2. Materials and Methods

### 2.1. Acquisition of Sylvirana latouchii Skin Secretions

Adult broad-folded frogs, *Sylvirana latouchii* (sex undetermined, *n* = 5, 3–5 cm snout-vent length) were captured in Fujian Province of China and then settled into a purpose-designed amphibian facility at 18–25 °C under 12 h/12 h light/dark cycled period. Skin secretion was collected by gentle transdermal electrical stimulation as the previous report [25] and rinsed off from the skin using deionised water. After lyophilisation, skin secretions were stored at −20 °C for later use.

The study was performed according to the guidelines in the UK Animal (Scientific Procedures) Act 1986, project license PPL 2694, issued by the Department of Health, Social Services and Public Safety, Northern Ireland. Procedures had been vetted by the IACUC of Queen’s University Belfast and approved on 1 March 2011.

### 2.2. “Shot-gun” Cloning of SL-BBI Precursor-Encoding cDNA

The biosynthetic precursor cDNA of SL-BBI was obtained as previously described [25]. Briefly, the Dynabeads^®^ mRNA DIRECT^TM^ Kit (Dynal Biotech, Merseyside, UK) was used to isolate the mRNA from the lyophilised skin secretion. The first-strand cDNA was synthesised by the BD SMART^TM^ RACE cDNA Amplification Kit (BD Bioscience Clontech, UK) and used to construct the cDNA library through 3′ RACE-PCR. For SL-BBI, the 3′-RACE reactions employed a NUP primer (supplied with the kit) and a degenerate sense primer (3′-RACE: 5′-GAWYYAYYHRAGCCYAAADATGTTCA-3′, R = A/G, V = A/C/G, N = A/C/T/G, Y = C/T, S = C/G, W = A/T) was designed based on the highly conserved nucleic acid sequences of the 5′-untranslated region of the protease inhibitory peptide published previously. The purified products were cloned by pGEM^®^-T Easy Vector Kit (Promega Corporation, Madison WI, USA) and sequenced through an ABI3730 automated sequencer (Applied Biosystems, Foster City, CA, USA).

### 2.3. Isolation and Structural Analysis of SL-BBI

Lyophilised skin secretions after the dissolution were used for isolation and identification of SL-BBI through a reverse-phase high-performance liquid chromatography (HPLC) fractionation system (Waters, Milford, MA, USA) over 240 min as previously reported [25]. Each fraction was analysed by a matrix-assisted laser desorption ionisation time-of-flight (MALDI-TOF) mass spectrometer (Voyager DE, PerSeptive Biosystems, Foster City, CA, USA), and the structure of selected peptide was confirmed by an LCQ-Fleet electrospray ion-trap mass spectrometer (Thermo Fisher Scientific, San Jose, CA, USA) through MS/MS fragmentation.

### 2.4. Solid-Phase Peptide Synthesis of SL-BBI and Its Analogues

After the confirmation of the sequence of the novel peptide from the translation of cloned cDNA and analyse of skin secretion samples, SL-BBI and two designed analogues (K-SL and F-SL) were chemically synthesised by Fmoc solid-phase peptide synthesis using a Tribute^®^ peptide solid-phase synthesiser (Protein Technologies, Inc, Tucson, AZ, USA) as previously reported [25]. The synthetic peptides were subjected to functional assessment after purified and identified by RP-HPLC and MALDI-TOF mass spectrometry.

### 2.5. Secondary Structure Analysis through Circular Dichroism (CD)

The secondary structure of SL-BBI and its analogues in 10 mM ammonium acetate and 50% TFE in 10 mM ammonium acetate buffer respectively were detected by a JASCO J-815 CD spectrometer (Jasco, Essex, UK) across the wavelength range of 190–250 nm and predicted by an online software BeStSel (http://bestsel.elte.hu) according to the previous report [25].

### 2.6. Trypsin and Chymotrypsin Inhibition Assay

Ten microliters of trypsin/chymotrypsin (Sigma Aldrich, Dorset, UK) working solution (0.1 µM in 1 mM HCl) was added into the wells of a black micro-titre plate with corresponding substrate and peptide replicates (0.1–100 µM) in 10 mM phosphate buffer (final volume 210 μL). Additionally, Phe-Pro-Arg- NHMec (Bachem, Cambridge, UK) and Succinyl-Ala-Ala-Pro-Phe-NHMec (Bachem, Cambridge, UK) were performed as substrates for trypsin and chymotrypsin, respectively. The fluorescence intensity of NHMec was monitored at 37 °C continuously for 30 min by a FLUOstar OPTIMA multi-well plate reader (BMG Labtech, Ortenberg, Germany) at wavelengths of 460 nm for emission and 395 nm for excitation. The inhibition curves of different protease were plotted using the Morrison equation and non-linear regression analysis, which were showed as outlined before [26].

### 2.7. Minimum Inhibitory Concentration (MIC) and Minimum Bactericidal Concentration (MBC) Assays

Antimicrobial activity of the synthetic peptides was assessed by determination of MIC/MBC on *Escherichia coli* (ATCC 11775), *Staphylococcus aureus* (ATCC 12600), *Candida albicans* (NCYC 1467) and methicillin-resistant *Staphylococcus aureus* (MRSA) (NCTC 12493) according to the previous report [25]. Briefly, the test microorganism was inoculated in Mueller Hinton Broth (MHB) medium and incubated overnight at 37 °C. Then the subculture was performed until the optical density (OD) reached the logarithmic growth phase and diluted to 1 × 10^6^ colony forming units (CFU)/mL for bacteria and 1 × 10^5^ CFU/mL for fungus obtained. The synthetic peptides with the concentration from 1 to 512 µM were double diluted by PBS. Five replications of sterile control, vehicle control and growth control were set as 100 µL MHB/well, 100 µL 1% PBS with subculture solution/well and 100 µl subculture solution/well, respectively. The 96-well plate was incubated at 37 °C overnight. The absorbance of each well at 550 nm was measured by Synergy HT plate reader (Biotech, Minneapolis, MN, USA). The minimum bactericidal concentration was detected by sub-culturing treated samples onto Mueller-Hinton agar (MHA) plate for another eighteen hours at 37 °C.

### 2.8. MTT Cell Viability and LDH (Lactate Dehydrogenase) Cytotoxicity Assay

MTT (3-(4, 5-dimethylthiazol-2-yl)-2, 5-diphenyltetrazolium bromide) cell viability assay was performed to evaluate the cell proliferation inhibitory rate of synthetic peptides in a series of human cell lines: PC-3 (ATCC^®^ CRL-1435™), H157 (RRID: CVCL_0463), H460 (ATCC^®^ HTB-177™), H838 (ATCC^®^ CRL-5844™), H23 (ATCC^®^ CRL-5800™), MCF-7 (ATCC^®^ HTB-22™), HCT116 (ATCC^®^ CCL-247™), U251MG (ECACC-09063001) and HMEC-1 (ATCC^®^ CRL-3243™) which was detailed in the previous report [5].

Pierce LDH Cytotoxicity Assay Kit (Thermo Fisher Scientific, Loughborough, UK) was used to detect the amount of LDH released from H157, H460, H1838 and H23 cells. 1 × 10^4^ cell per 100 µL of final cell suspension was deemed to be ideal in each well of a 96-well plate. After 24 h incubation (37 °C, 5% CO^2^), vehicle control and different concentration of sample solution were loaded on the plate which was further incubated for 24 h. 10 µL 10× Lysis Buffer was added as Maximum LDH Activity control for 45 min and 10 µL water was added as Spontaneous LDH Activity control for the same time. 50 µL aliquots of each well were transferred to a well of a fresh 96-well plate and mixed with 50 µL of Reaction Mixture. After incubation for 30 min (room temperature, lightless), stop solution (50 µL/well) was added to stop the reaction. The absorbance values at 490 nm and 680 nm were measured by Synergy HT plate reader (Biotech, Minneapolis, MN, USA).

### 2.9. Haemolysis Activity Assay

4% (*v*/*v*) suspensions of horse erythrocytes (TCS Biosciences, Botolph Claydon, Buckingham, UK) in phosphate-buffered saline (PBS) was used to evaluate haemolysis activity by incubating with different concentration of synthetic peptides from 1 µM to 512 µM at 37 °C for 2 h as detailed in the previous report [5].

### 2.10. Cells Apoptosis Detection

Cell apoptosis was determined by using Muse™ Annexin V & Dead Cell Reagent (EMD Millipore, Billerica, MA, USA) according to the manufacturer’s instructions. H157 cells were seeded at 1.5 × 10^5^ cells/mL in a 12-well plate and incubated overnight in a humidified atmosphere containing at 37 °C under 5% CO_2_. After which, cells were treated with 1, 10 and 100 µM F-SL for 8 h. Positive control groups were treated with 200 µM carboplatin for the same time. After treatment, the growth medium was removed and cells washed with ice-cold PBS twice and centrifuged at 300× *g* for 3 min at 4 °C and re-suspended by growth medium. Cell suspension (100 μL) was stained with 100 μL Muse Annexin V & Dead Cell Reagent for 20 min at room temperature protect from light and the suspension was analysed using Muse Cell Analyzer (EMD Millipore, Billerica, MA, USA).

### 2.11. Caspase 3/7 Activity Assay

Caspase 3/7 activity was determined by using MUSE™ Caspase-3/7 Kit (EMD Millipore, Billerica, MA, USA) according to the manufacturer’s instructions. For the caspase 3/7 activity detection, H157 cells were seeded at 1.5 × 10^5^ cells/mL in a 12-well plate and allowed to attach overnight in a humidified atmosphere containing 5% CO_2_ at 37 °C. After which, cells were treated with 1, 10 and 100 µM peptide for 24 h. Positive control groups were treated with 200 µM carboplatin for the same time. After treatment, growth medium was removed and cells were twice washed with ice-cold PBS and centrifuged at 300× *g* for 3 min at 4 °C and re-suspended by 1 × assay buffer, stained with MUSE Caspase-3/7 reagent for 30 min at 37 °C. Then mixed the cell with the 7-AAD reagent and analysed using Muse Cell Analyzer (EMD Millipore, Billerica, MA, USA).

### 2.12. Modelling and Molecular Docking Analysis

The peptide sequences of SL-BBI and its analogue obtained by molecular cloning and structural identification were submitted to the I-TASSER server (https://zhanglab.ccmb.med.umich.edu/I-TASSER/) for three-dimensional (3D) structure modelling [27]. The 3D structure of the yeast 20S proteasome (PDB ID: 2F16) comes from the protein database bank (PDB) (https://www.rcsb.org/) and the targeted subunits were separated. To simulate the main binding conditions between the ligand peptide (F-SL) and the receptor protein (β1 and β5 subunits) with a known 3D structure, the water molecules and the original ligand in the structure of the candidate docking complex were eliminated. Then the Z-DOCK server (http://zdock.umassmed.edu/) was used to perform rigid molecular docking on all receptor molecules and ligand. ZDOCK will search all the translation and rotation spaces of the receptor and ligand molecules and then use energy-based functions to score each possible pose, which includes potential energy, space complementation and electric field force in the calculations. [28,29]. The highest-ranked ZDOCK pose was selected and Accelrys Discovery Studio software (Biovia, San Diego, CA, USA) was used for the visualization of interaction structures.

### 2.13. Statistical Analysis

Each data point averaged by three independent experiments and expressed as means ± standard error (SEM). All of the data were statistically analysed by Prism 6 (GraphPad Prism Software, GraphPad, SanDiego, CA, USA) and one-way analysis of variance (ANOVA) of the differences. *p* < 0.05 was considered to be significant.

## 3. Results

### 3.1. Identification and Structural Characterisation of SL-BBI

**Figure 1:**
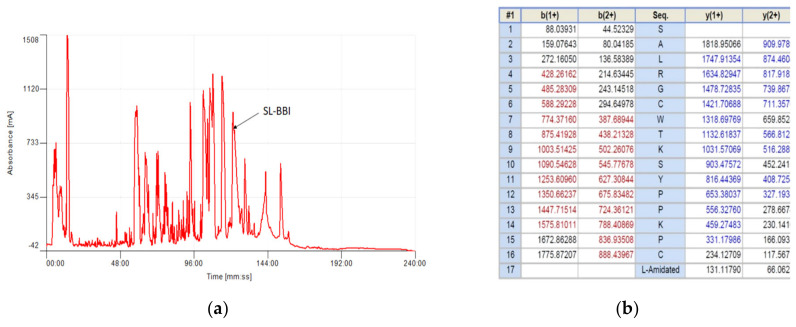

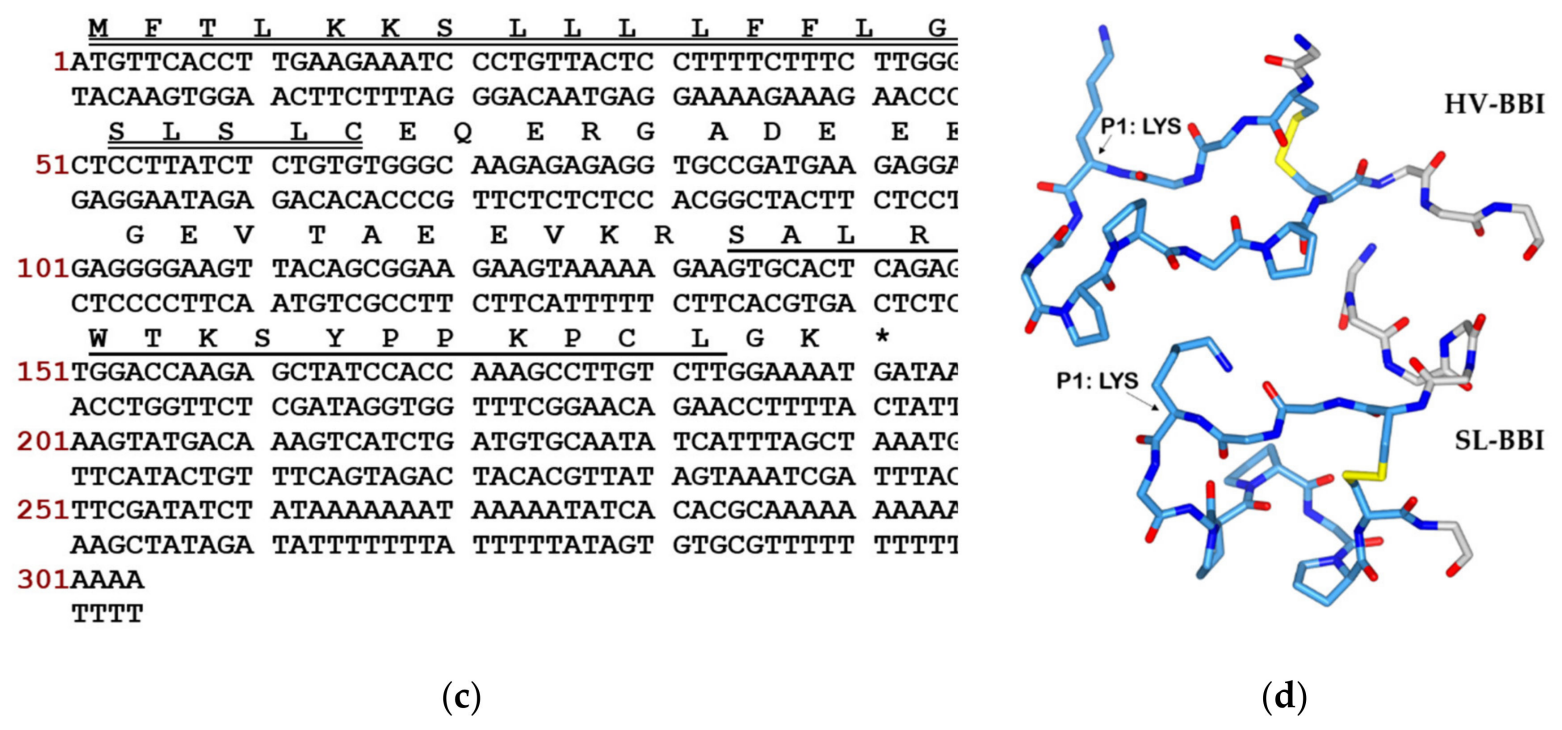
Identification and structural features of SL-BBI. (**a**) Region of RP-HPLC chromatogram of *Sylvirana latouchii* skin secretion with the elution position of the SL-BBI indicated by a labelled arrow. (**b**) Electrospray ion-trap MS/MS fragmentation data derived from fragment ions corresponding in molecular mass to SL-BBI. Predicted singly and doubly charged b-ions and y-ions are in black typeface. Ions detected by MS/MS fragmentation are indicated in red and blue. (**c**) Nucleotide and translated amino acid sequence of SL-BBI which cloned from the skin secretion-derived library of *Sylvirana latouchii*. The putative signal sequences are double-underlined, the mature sequences are single-underlined and an asterisk indicates the stop codon. (**d**) Comparison of the structure of HV-BBI and simulated SL-BBI in stick model (CPK colouring, with carbon atoms inside the canonical loop in light blue and outside in grey). The LYS residue at the P1 site was additionally annotated.

**Figure 2:**
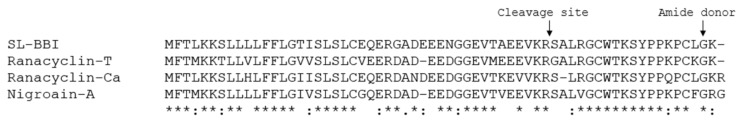
Alignments of partial translated amino acids sequences of Ranacyclin peptides. Ranacyclin T, Ranacyclin Ca and Nigroain-A were identified from *Rana temporaria*, *Lithobates catesbeianus* and *Sylvirana nigrovittata*, respectively [2,30]. Amino acid sequences aligned in ClustalW format were analysed by Clustal W2 multiple sequence alignment program. Asterisk, colons and periods indicate the conservation of residues from high to low.

The cDNA encoding the biosynthetic precursor of a trypsin inhibitory peptide was successfully cloned from the skin secretion-derived cDNA library of *Sylvirana latouchii* and was named SL-BBI. After fractionated of lyophilised skin secretions by RP-HPLC, the molecular mass of each fraction was identified by MALDI-TOF MS and the elution site of SL-BBI was confirmed (Figure 1a). The primary structure of this novel peptide was subsequently confirmed by MS/MS fragmentation sequencing (Figure 1b and Figure S1). The open reading frame of SL-BBI was made up of 192 base pairs encoding 63 amino acids (Figure 1c). The putative signal peptide was composed of 22 amino acids and the mature peptide with 17 amino acids was processed at a classical propeptide convertase cleavage site (-KR-) following an acidic residue rich residue. Simulated structure of SL-BBI in stick model showed a similar configuration to peptide HV-BBI (PDB ID: 4U2W) which belongs to the Ranacyclin family (Figure 1d). The sequence of the mature peptide was searched and analysed by the Basic Local Alignment Search Tool (BLAST) program on NCBI and showed obvious similarity with amphibian-derived Bowman–Birk type inhibitory peptides (Figure 2). The nucleotide sequence of cDNA precursor of SL-BBI was deposited in the Genbank Database under an accession number: MT756002.

### 3.2. Motif-Targeted Peptide Design and Synthesis

Two analogues K-SL and F-SL were designed and synthesised according to the wild structure of SL-BBI with a disulphide bond formed between C6 and C16 (Table 1). Molecular mass of three synthetic peptide were observed by MALDI-TOF mass spectrometry in Figure S2. The purpose of the modification of K-SL is to promote its antimicrobial activity. The substitution of alanine (A) and lysine (K) at the head and tail increased the net positive charge and enhanced its selectivity for negatively charged bacterial cell membranes. The P1 site within the active disulphide loop of the peptides from BBI family is considered to be the most critical marker for binding to the specific S1 substrate pocket of the target enzyme, which located between the highly conserved Thr and Ser residues. The substitution of phenylalanine (F) in P1 site within the active disulphide loop was designed to enhance its chymotrypsin inhibitory and antiproliferative activity and showed the higher GRAVY than SL-BBI. The mass and purity of the synthetic peptides were determined by MALDI-TOF MS and RP-HPLC.

### 3.3. Secondary Structure Prediction of SL-BBI and Its Analogues

**Figure 3:**
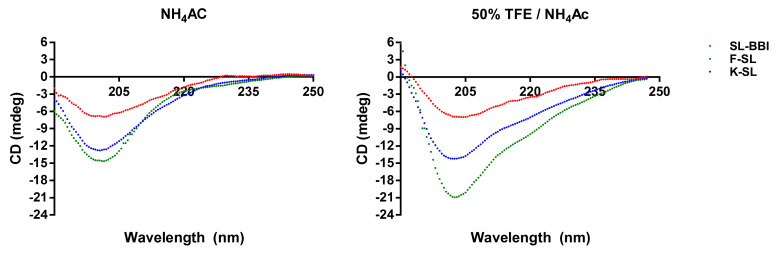
Circular dichroism spectra of SL-BBI (coloured red), K-SL (coloured green) and F-SL (coloured blue) (100 µM) in aqueous ammonium acetate buffer and membrane mimic 50% TFE/10 mM ammonium acetate buffer.

The secondary structure was analysed by using CD spectroscopy. SL-BBI and its analogues formed similar secondary structures in 50% TFE/10 mM ammonium acetate buffer and 10 mM ammonium acetate buffer (Figure 3). The percentage of secondary structure type of different peptide in each solvent environment was calculated (Table 2). See the secondary structure of these peptides are mostly composed of β-sheet and random coil in the neutral solvent. However, there is an increase of the helix structure of K-SL in the simulated hydrophobic cell membrane environment.

### 3.4. Trypsin and Chymotrypsin Inhibitory Activity

**Figure 4:**
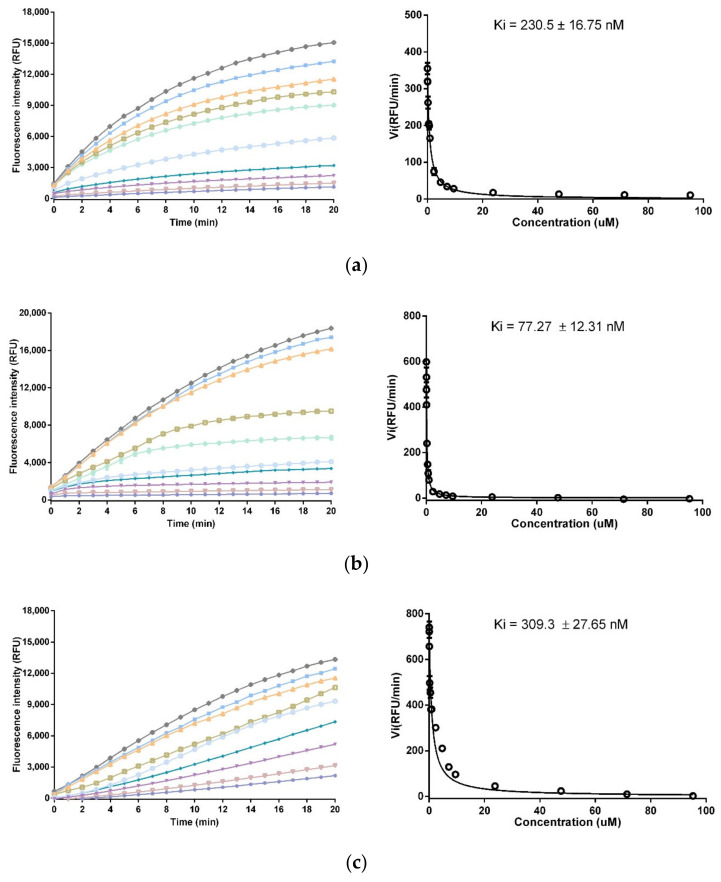
Inhibitory effect of SL-BBI, K-SL and F-SL on trypsin and chymotrypsin. (**a**) Progress curves for trypsin proteolysis under SL-BBI and corresponding Morrison Ki plot performed via final steady-state rates (Vi). (**b**) Progress curves for trypsin proteolysis under K-SL and corresponding Morrison Ki plot. (**c**) Progress curves for chymotrypsin proteolysis under F-SL and corresponding Morrison Ki plot. Data points were fitted to the curve by non-linear regression analysis, using GraphPad Prism. (⬤ 23.81 μM, ▽ 7.143 μM, ▼ 4.762 μM, ◆ 2.381 μM, ◯ 0.9542 μM, ◆ 0.7143 μM, ◻ 0.4762 μM, △ 0.2381 μM, ◼ 0.02381 μM, ◆ 0 μM).

The inhibition effects were evaluated through trypsin and chy±motrypsin. The substrate hydrolysis progress curve and corresponding Morrison Ki plot were shown in Figure 4. SL-BBI exhibited potent inhibition activity to trypsin with a Ki value of 230.5 ± 16.75 nM while K-SL showed promote efficiency with a Ki value of 77.27 ± 12.31 nM. The substitution of Phe in P1 position conferred F-SL on chymotrypsin inhibitory effect with a Ki value of 309.3 ± 27.65 nM but lost the trypsin inhibitory activity.

### 3.5. Antimicrobial and Haemolytic Activity

**Figure 5:**
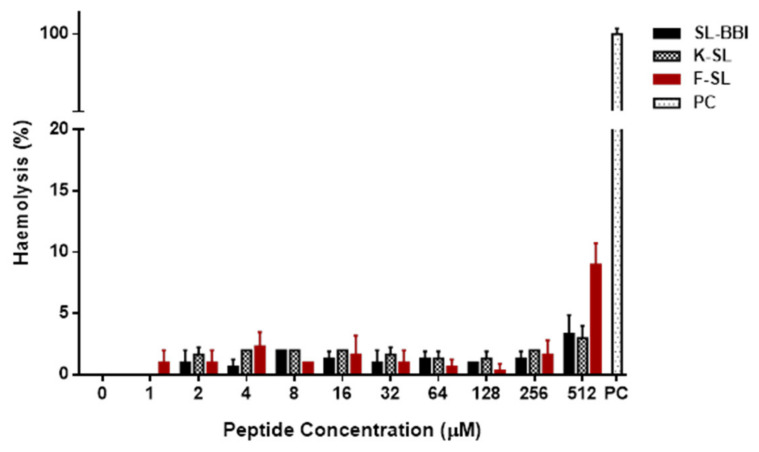
The hemolytic effect of SL-BBI, K-SL and F-SL against horse erythrocytes after 2 h incubation. The positive control group was incubated with 1%(*v*/*v*) Triton X-100. The error bar represents the S.E.M. (standard error of the mean) of 5 replicates.

The inhibition capacities of SL-BBI, K-SL and F-SL on representative microorganisms were shown in Figure S3 and Table 3. Compared with SL-BBI, K-SL performed comprehensive improvement in the inhibitory capability against *S. aureus* and *C. albicans* but no promotion in *E. coli* and MRSA. However, F-SL completely lost antimicrobial activity. All three peptides maintained low haemolytic activity in horse erythrocytes (Figure 5).

### 3.6. Anti-Proliferation Activity on Human Cancer and Normal Cells

**Figure 6:**
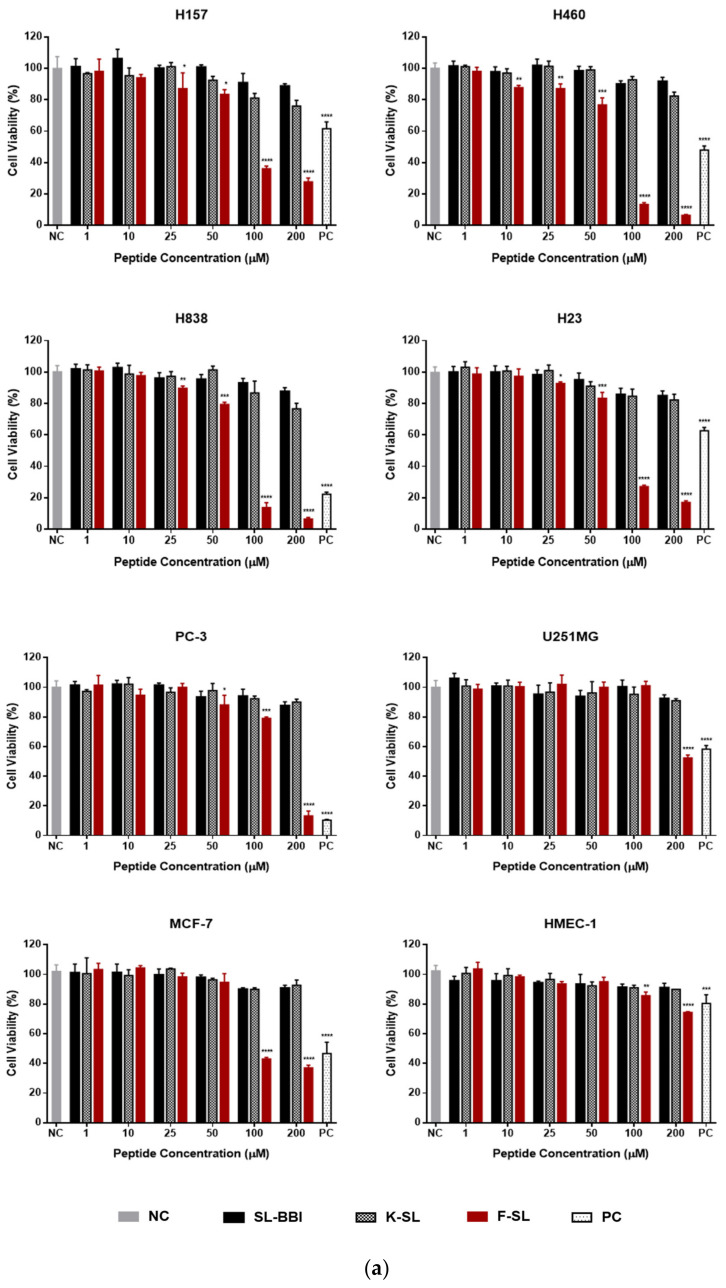

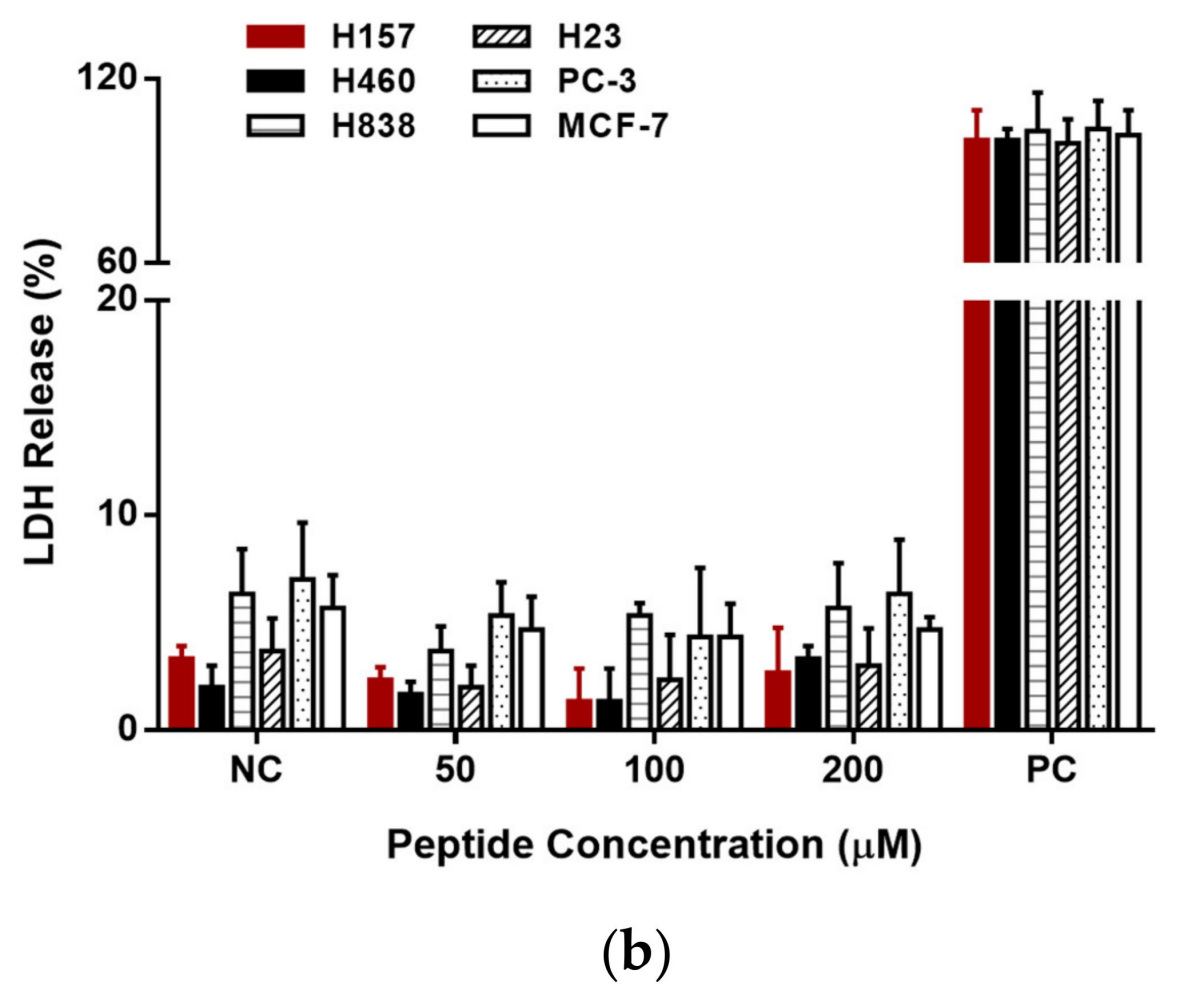
Cytotoxicity evaluation of SL-BBI and its analogues on different cell lines. (**a**) The MTT cell viability test on H157, H460, H838, H23, PC-3, U251MG, MCF-7, HCT-116 and HMEC-1 cells under different concentration after 24 h treatment. The 100% cell viability was applied with growing cells without peptides and a positive control group was treated with 1 mM 5-FU. (**b**) The LDH cytotoxicity results of F-SL on H157, H460, H838, H23, PC-3 and MCF-7. The 100% LDH release of the positive control group was applied with 1% Triton X-100 and negative control group was acquired after treated with culture medium. The error bar represents the S.E.M. (standard error of the mean) of 9 replicates and the levels of significance are: * *p* < 0.05, ** *p* < 0.01, *** *p* < 0.001, **** *p* < 0.0001.

SL-BBI, K-SL and F-SL were subjected to MTT cell viability assays on series of human non-small cell lung cancer (NSCLC) cell lines (H157, H460, H838 and H23) and other cancer cell lines (PC-3, U251MG and MCF-7). As shown in Figure 6a, chymotrypsin inhibitor F-SL exhibited reinforced anti-proliferation effect on PC-3 and MCF-7 compared with other two trypsin inhibitors, especially on a series of NSCLC cells. Its toxicity to HMEC-1 was mildly enhanced but remained at a slight level at tested concentrations. The IC50 value of F-SL in Table 4 represented mean ± S.D. (standard deviation) calculated from 3 independent experiments. Correspondingly, LDH release level under sufficient concentrations was tested as the index of cell membrane integrity. Contrary to the MTT results, F-SL did not show the ability to lyse cell membranes in any kind of cancer cells (Figure 6b).

### 3.7. F-SL Exerts Anti-Cancer Effect by Apoptosis

To determine the mechanism of cell deaths, the Annexin V/7-AAD and Caspase 3/7 cell apoptosis detection were carried out on F-SL-treated H157 and H838 cells by flow cytometry. The phosphatidylserine (PS) present on the inner surface of the cell membrane outward to the surface is a common phenomenon in early and late apoptotic cells. Annexin V is a Ca+-dependent binding protein with high affinity for PS, which is often used in combination with nucleic acid dyes to distinguish live cells, apoptotic cells and dead cells.

**Figure 7:**
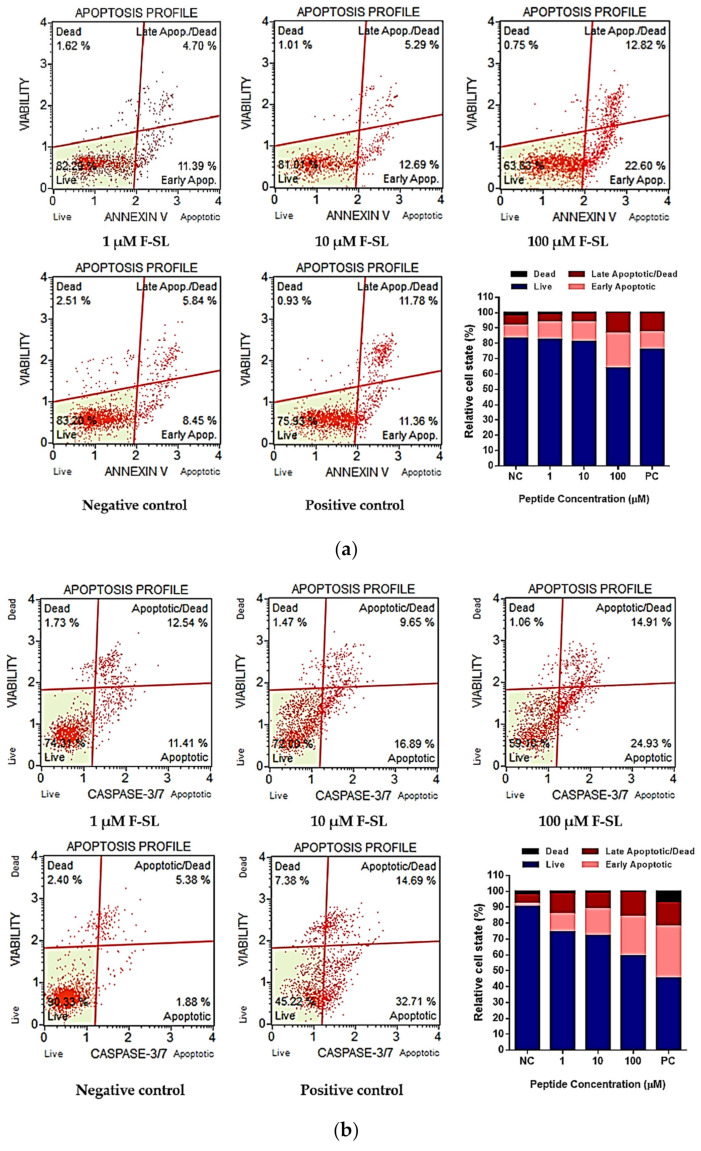

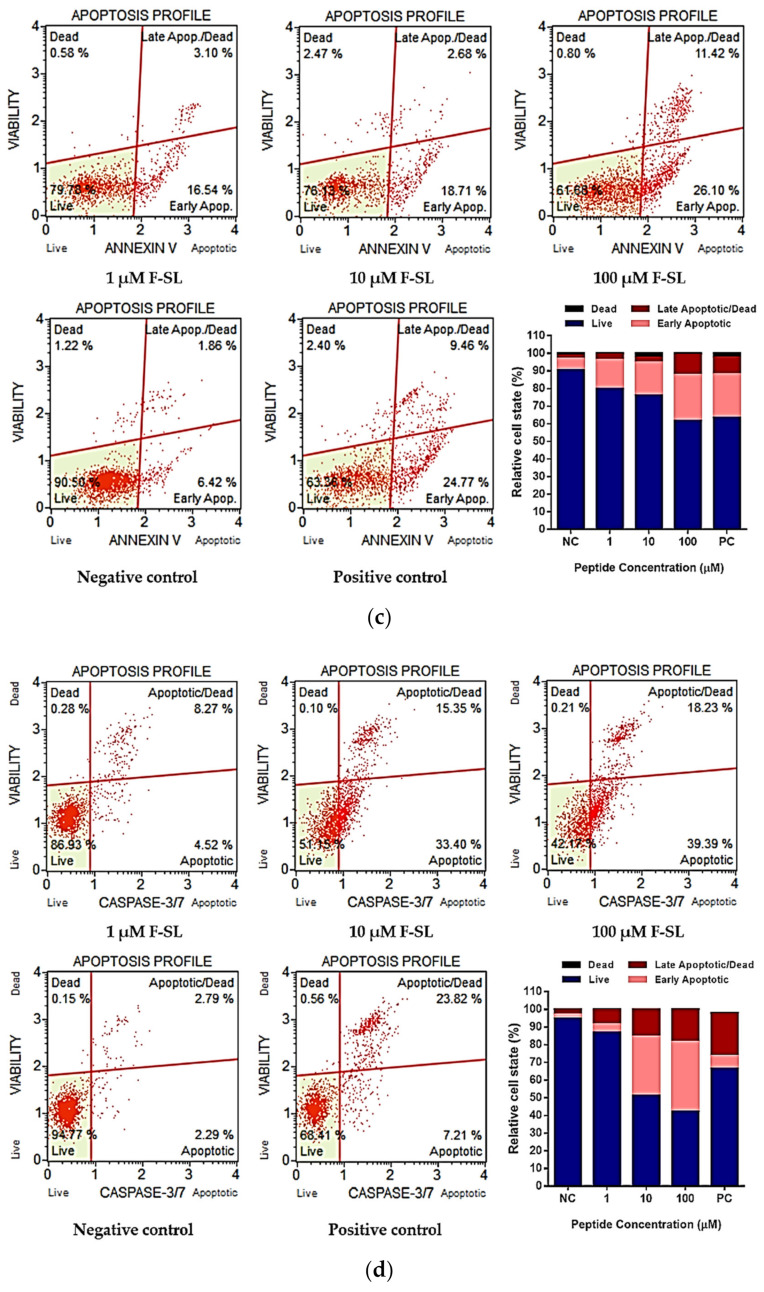
Assessment of apoptosis and relative state percentage in H157 and H838 cell lines induced by F-SL of different concentrations. (**a**) Representative plots from Annexin V-FITC/7-AAD assay after 8 h incubation on H157. (**b**) Representative plots from Caspase 3/7/7-AAD assay 16 h incubation on H157. (**c**) Representative plots from Annexin V-FITC/7-AAD assay after 8 h incubation on H838. (**d**) Representative plots from Caspase 3/7/7-AAD assay 16 h incubation on H838. The gated cells with quadrant marker divided date into live, apoptotic, apoptotic/dead and dead populations. Positive drug control groups were treated with 1 mM 5-FU and negative control groups were acquired after treated with culture medium.

Compared with the non-treated control group, F-SL treatment significantly increase the percentage of H157 and H848 cells in Annexin V (+)/7-AAD (−) and Annexin V (+)/7-AAD (+) categories (Figure 7a,c). For H157, total apoptotic cells increased to 16.09% (1 μM), 17.98% (10 μM) and 35.42% (100 μM) from 14.29% (negative control) after 8h incubation. Similarly, the results of Caspase 3/7 activity test showed that F-SL can induce the increased the percentage of H157 cells in caspase 3/7 (+)/7-AAD (−) and caspase 3/7 (+)/7-AAD (+) categories (Figure 7b), which increased total apoptotic cells from 7.26% (negative control) to 23.95% (1μM), 26.54% (10 μM) and 39.84% (100 μM). For H838, total apoptotic cells increased to 19.64% (1 μM), 21.39% (10 μM) and 37.52% (100 μM) from 8.28% (negative control) after 8 h incubation. Correspondingly, the results of Caspase 3/7 activity test indicated that F-SL can induce the increased the percentage of H838 cells in caspase 3/7 (+)/7-AAD (−) and caspase 3/7 (+)/7-AAD (+) categories (Figure 7d), which increased total apoptotic cells from 5.08% (negative control) to 12.79% (1 μM), 48.75% (10 μM) and 57.62% (100 μM). The assessment of apoptosis by Annexin V and Caspase3/7 assay indicated that F-SL leads to programmed death of H157 and H838 cells in a dose-dependent manner.

### 3.8. Molecular Docking Simulation of F-SL

**Figure 8:**
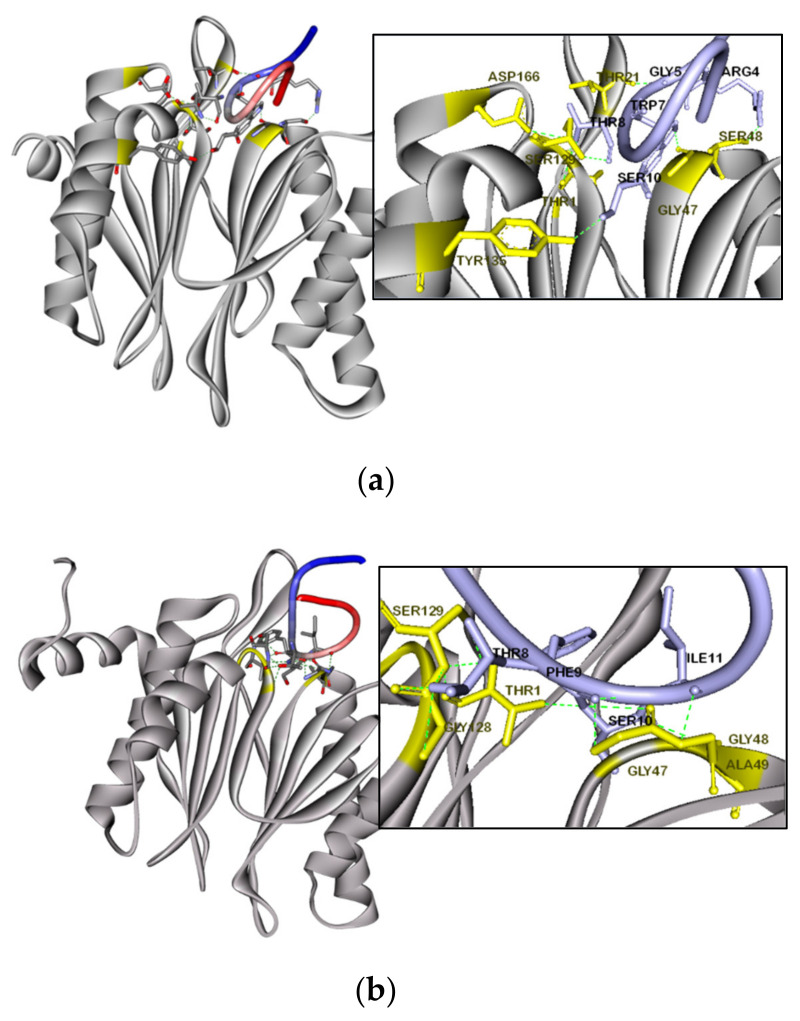
Simulated interaction of the protease-inhibitor docking for F-SL with β1 (**a**) and β5 (**b**) subunits of yeast 20S proteasome. The residues involved in the interaction on the corresponding subunits of F-SL (N-terminus is blue and C-terminus is red) were shown in stick representation. Possible active binding sites in subunits were highlighted in yellow and the hydrogen bonds were represented by green dotted lines.

Accelrys Discovery Studio software was used to evaluate the amino acids and their molecules involved in H-bonding in the predicted docking model generated by the ZDOCK online server. ZDOCK obtains all possible mutual binding modes of β1/β5 subunits in F-SL and yeast 20S proteasome in space through shape complementation, and uses the energy-based scoring function to evaluate the reliability of each binding model. The most reliable models are shown in Figure 8. The simulation results showed that the Thr1, Thr21, Gly47, Ser48 and Tyr135 residues of the beta1 subunit receptor were expected to interact with the Thr8, Gly5, Trp7 and Ser10 residues in the F-SL ligand, respectively (Figure 8a). And the Thr1, Gly47, Gly48 and Ala49 residues of the β5 subunit receptor were expected to interact with Ser10, Phe9, Ile11 and Ser10 residues in the active loop of the F-SL ligand, respectively (Figure 8b). These results reflected our prediction of the mechanism of peptide binding from the side. F-SL may have a high affinity for the β1 and β5 subunits with catalytic effects in the 20S core particles, thereby affecting the related cancer pathological processes regulated by the proteasome pathway.

## 4. Discussion

The active substances in amphibian skin secretions have always occupied an essential position in the field of natural drug discovery. Currently, a variety of bioactive peptides of brevinin, temporin, ranatensin, nigrocin and ranacyclin families have been discovered from broad-folded frog (*Sylvirana latouchii*) [31,32,33,34] and showed potential medicinal value. Here, we found a novel Bowman–Birk type trypsin inhibitor peptide from this species named SL-BBI, which shared the highly conserved ‘-CWTP_1_SXXPKPC-’ ring with the ranacyclin family. In addition to structural and functional identification, we also made target-modifications to promote its antimicrobial and anticancer capabilities.

The ranacyclin family was previously regarded as a branch of antimicrobial peptides [2]. However, as more extensive discoveries and studies have shown that its ability to inhibit serine protease is more prominent than antimicrobial [11]. SL-BBI is undoubtedly a highly effective trypsin inhibitor with a Ki value of 230.5 nM. Due to its antimicrobial performance is not satisfactory, K-SL was designed by substitution of Ala, Ile and Lys in position 1, 11 and 17, respectively. Structural changes did not excessively affect its trypsin inhibitory effect and remained at a Ki value of 77.27 nM. It is well known that rich in positively charged amino acids is one of the characteristics of antimicrobial peptides [35], which means that trypsin-like proteases that regard Lys and Arg as cleavage sites are easy to hydrolyse them. The antimicrobial effect of Ranacyclin family peptides is attributed to the increase in permeability of cytoplasmic phospholipid membranes and the formation of transmembrane pores by inserting hydrophobic cores [2,13]. This mechanism of maintaining the integrity of the cell membrane is different from most antimicrobial peptides. The substituted Ile in P_2′_ site with large aliphatic side chain in the active disulphide loop provided optima packing to the apolar S_2_ substrate pocket of serine proteases and retarded hydrolysis rate [36], and the replacement of N-terminal and C-terminal ends amino acids enhanced the cationicity with reference to the sequences of ORB [37] and Ranacyclin-T/E [2]. The increase of positive charges and the α-helix structure formed in the simulated hydrophobic cell membrane environment may help K-SL act on the negative charged bacterial cell membrane. Correspondingly, K-SL showed the stronger antimicrobial effect to *S. aureus* and *C. albicans* with MIC value of 64 μM but maintained the same MIC value of 128 μM in *E. coli* with wild SL-BBI. On the contrary, the substitution of Phe at the P_1_ site (F-SL) resulted in a significant reduction in positive charge and loss of antimicrobial activity. In addition, SL-BBI and its analogues showed almost no haemolytic effect on horse erythrocytes even at a high concentration of 512 μM. This is similar to the characteristic that the cyclic pLR (belongs to Ranacyclin family) can produce strong membrane permeability for the electronegative POPG membrane but has no effect on the electrically neutral POPC membrane [38]. Some analysis pointed out that members of the Ranacyclin family with high-efficiency antibacterial activity may gather on the surface of bacterial cell membranes to form channel structures due to the strong hydrophobic phenylalanine exposed at the C-terminal [37]. The characteristic of rich in cationic residues in antimicrobial peptides predestined their sensitivity to trypsin-like proteases, which indicates that bifunctional peptides with trypsin-like inhibition and antimicrobial activities will become ideal candidates for anti-infective agents. Although the inhibitory effects are not produced in drug-resistant strains, bifunctional peptides with antimicrobial and protease inhibitory activities may become ideal candidates for a new generation of antibiotics, which can kill microorganisms while resisting degradation of proteases.

The anticarcinogenesis of BBI type inhibitors is generally considered to be more relevant with the proteasomal chymotrypsin-like activities [14,21,22], which also been proved by the discovery of HECI from the Asian green frog [5], a natural chymotrypsin inhibitor with antiproliferative effect. BBI and its concentrated products (BBIC) can not only enhance the killing effect of cisplatin on lung cancer and ovarian cancer cells and protect normal cells in vitro [39,40,41], but also inhibit the conversion of a series of carcinogens such as dimethylhydrazine and 3-methylcholanthrene in different organs in vivo [42,43]. The precise mechanism of protease inhibitors exhibiting anticancer activity is still widely debated, but the contribution of chymotrypsin inhibition probably indicates the influence in the ubiquitin-proteasome pathway (UPP) in tumor cells. UPP mediates transcriptional and apoptosis regulation by controlling crucial proteins such as the activation of caspases and NF-kB and the degradation of Bcl-2 [44,45]. As the core particle of mammalian 26 proteasomes in UPP, 20S proteasome consists of three major active subunits, β1, β2 and β5, which assigned to different activities to caspase-like, tryptic-like and chymotryptic-like by the nature of their S1 substrate pocket [5,46,47].

In this way, we designed F-SL for the distinction of specific substrate pocket of different serine proteases and used Phe at active P1 position to best match chymotrypsin-like subunit. Firstly, the substitution of Lys by Phe authentically resulted in an increase in chymotrypsin inhibitory effectiveness to a Ki value of 309.3 nM in F-SL but lost the trypsin inhibitory activity at the same time. The docking results of this study show an excellent superposition of the β1 and β5 subunits in yeast 20S proteasome with F-SL, which was similar to the covalent and reversible binding by classic proteasome inhibitor bortezomib with the Thr at the active sites of the β5 subunit [48]. As the first clinical proteasome inhibitor, bortezomib was originally used in the treatment of multiple myeloma, but recent studies have shown that it can be used alone or in combination therapy for pancreatic, prostate, breast or non-small cell lung cancers [49,50]. MTT cytotoxicity test suggested that chymotrypsin inhibitor F-SL is the most potent anticancer agent with antiproliferative effect in a series of non-small cell lung cancer cell lines (H157, H460, H838 and H23), which also showed a certain inhibitory effect on prostate cancer cell PC-3 and breast cancer cell MCF-7 while SL-BBI and K-SL were totally inactive. However, F-SL was unable to cause the release of LDH in cells at a concentration with antiproliferative activity, which indicated that it will not cause cell membrane rupture and contents leakage. PS eversion of the inner surface of the cell membrane is the most commonly used basis for judging cell apoptosis. According to the significant increase in Annexin V-FITC positive cell population, we found F-SL induced apoptosis in H157 and H838 cell lines in dose-dependent manner. Caspase 3 and caspase 7 are the most important executioners in the cysteine-aspartic acid protease (caspase) family involve in cell apoptosis cascade which possess similar substrate specificity. Caspase 3/7 zymogens are inactive until hetero-activation is performed by the upstream initiator caspase 8 after apoptotic signal delivered and then degrade the cellular components in a controlled manner [51]. Therefore, F-SL-induced caspase 3/7 activation in H157 and H838 cells confirmed its induction of apoptosis. We conjectured that F-SL may have a similar mechanism of apoptosis induction as Bortezomib: inhibit the activity of proteasomes in cells and thus preventing the activation of NF-kB and Bcl-2 to enhance the cascade activation of caspase [47,52,53]. This inhibitory effect can also cause subsequent mitochondrial impairment and oxidative damage [14]. On the other hand, the anti-proliferative effect of F-SL on cells appears to be selective against cancer cells. Not only is it almost non-toxic to horse erythrocytes, but it also showed less antiproliferative effect on normal human endothelial cells HMEC-1. This difference may cause by abnormal protein activity in malignant cells with high proliferation. Tumor cells are more sensitive to relative inhibitors because they rely more on UPP to degrade abnormal proteins or even reverse cell cycle and apoptosis checkpoint mutations to cause tumorigenesis [47].

In summary, SL-BBI is a novel peptide with bifunction of potent trypsin inhibitory and weak antimicrobial from the skin secretion of *Sylvirana latouchii* which belongs to the ranacylin family. Through targeted structural modification, two analogues K-SL and F-SL with improved antimicrobial and anticancer activity were obtained respectively. F-SL with chymotrypsin inhibitory activity exhibits targeted induction of cancer cell apoptosis which involved caspase 3/7 activation. It also shows that the short cyclic peptide of the ranacyclin family is an excellent template for the development of multifunctional peptides and provide more drug candidates for cancer chemotherapy and prevention in the future.

## Figures and Tables

**Table 1:** Sequences and physicochemical properties of SL-BBI and its designed analogues.

Peptides	Sequence	Net Charge	GRAVY ^1^	Theoretical Mass (Da)	Observed Mass (Da)
SL-BBI	SALRGCWT**K**SYPPKPCL-NH_2_	+4	−0.447	1904.34	1904.60
K-SL	AALRGCWT**K**SIPPKPCK-NH_2_	+5	−0.406	1853.33	1851.88
F-SL	SALRGCWT**F**SIPPKPCL-NH_2_	+3	0.288	1873.32	1873.82

^1^ GRAVY: Grand average of hydropathicity.

**Table 2:** The proportion of different secondary structures domain (%) was predicted by using the online software, BeStSel (Beta Structure Selection).

Peptides	NH_4_AC			50%TFE/NH_4_AC		
	Helix	Antiparallel	Others	Helix	Antiparallel	Others
SL-BBI	13	33	54	23	32	45
K-SL	15	38	47	29	26	45
F-SL	13	40	47	13	36	51

**Table 3:** Antimicrobial effect of SL-BBI and its analogues against tested microorganisms.

Peptides	MIC/MBC (μM)			
	*S. aureus*	*E. coli*	*C. albicans*	MRSA
SL-BBI	256/256	128/128	256/256	>512
K-SL	64/64	128/128	64/64	>512
F-SL	>512	>512	>512	>512

**Table 4:** The calculated IC50 values of F-SL on different human cell lines based on MTT results.

Peptides	IC50 (μM)						
	H157	H460	H838	H23	PC-3	MCF-7	HMEC-1
F-SL	101.4 ± 1.74	65.99 ± 2.64	59.74 ± 2.72	79.06 ± 6.41	158.6 ± 6.44	201.7 ± 10.6	573.5 ± 9.41

## Supplementary Materials

The following are available online at https://www.mdpi.com/2218-273X/10/9/1254/s1, Figure S1: MS/MS fragmentation data derived from fragment b- and y-ions corresponding in molecular mass to SL-BBI, Figure S2: MALDI-TOF mass spectrums of synthetic peptide SL-BBI (a), K-SL (b) and F-SL (c), Figure S3: Inhibitory effects following incubation of SL-BBI, K-SL and F-SL with S. aureus, MRSA, C. albicans and E. coli in a range of concentrations from 1 μM to 512 μM.
