# Supplementary material for: Identification and Target-Modification of SL-BBI: A Novel Bowman–Birk Type Trypsin Inhibitor from Sylvirana latouchii
Source: Biomolecules. 2020 Aug 28;10(9):1254. doi: 10.3390/biom10091254 (PMC7565067; doi:10.3390/biom10091254)
Supplement: Supplementary file 1 [file biomolecules-10-01254-s001.pdf]

Identification and Target-modification of SL-BBI: A Novel Bowman-Birk Type Trypsin Inhibitor from *Sylvirana latouchii*

Supplement materials

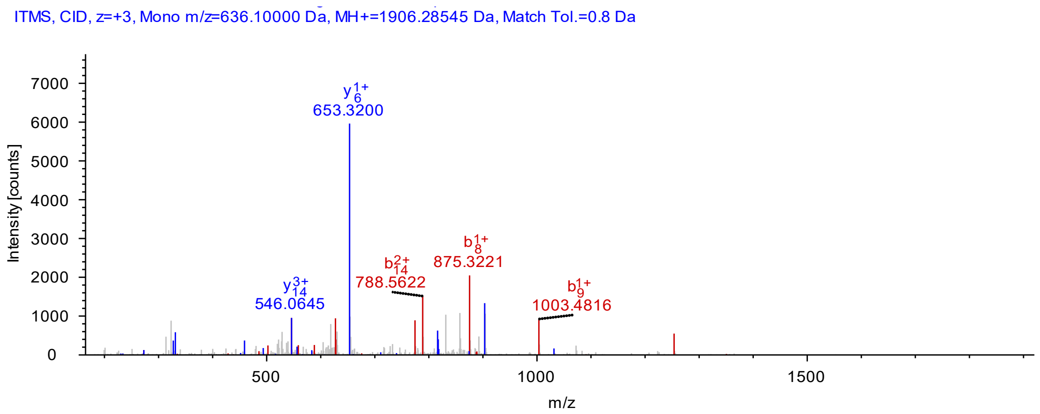

Figure S1 MS/MS fragmentation data derived from fragment b- and y-ions corresponding in molecular mass to SL-BBI.

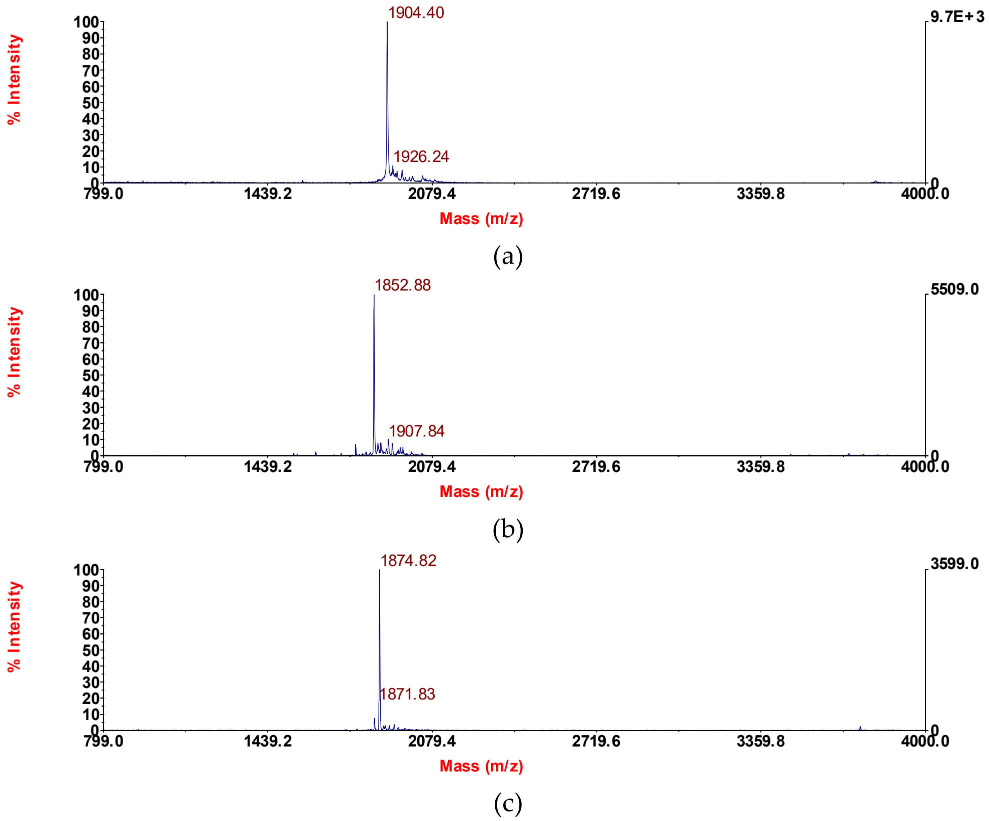

Figure S2 MALDI-TOF mass spectrums of synthetic peptide SL-BBI (a), K-SL (b) and F-SL (c).

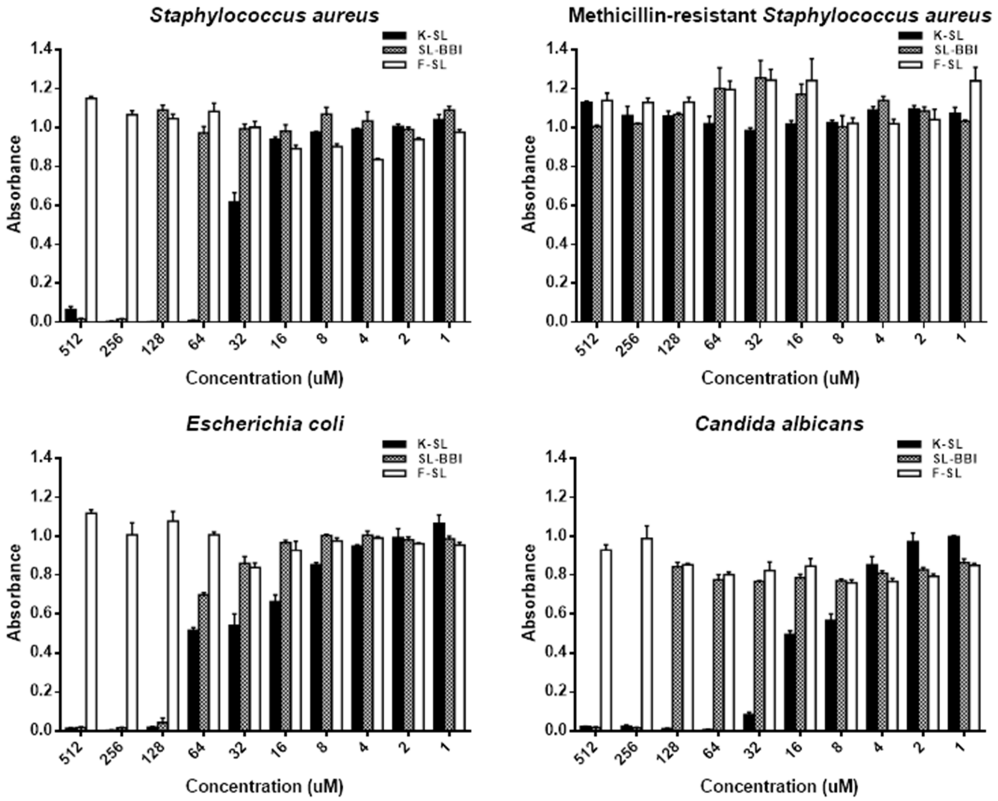

Figure S3 Inhibitory effects following incubation of SL-BBI, K-SL and F-SL with *S. aureus*, MRSA, *C. albicans* and *E. coli* in a range of concentrations from 1 μM to 512 μM. The error bar represents the S.E.M. (standard error of the mean) of 15 replicates.
